# Impact of maternal vaccination timing and influenza virus circulation on birth outcomes in rural Nepal

**DOI:** 10.1002/ijgo.12341

**Published:** 2017-11-09

**Authors:** Naoko Kozuki, Joanne Katz, Janet A. Englund, Mark C. Steinhoff, Subarna K. Khatry, Laxman Shrestha, Jane Kuypers, Luke C. Mullany, Helen Y. Chu, Steven C. LeClerq, James M. Tielsch

**Affiliations:** ^1^ Department of International Health Johns Hopkins Bloomberg School of Public Health Baltimore MD USA; ^2^ Seattle Children's Hospital and Research Foundation University of Washington Seattle WA USA; ^3^ Department of Pediatrics Cincinnati Children's Hospital Medical Center Cincinnati OH USA; ^4^ Nepal Nutrition Intervention Project – Sarlahi Kathmandu Nepal; ^5^ Department of Pediatrics and Child Health Institute of Medicine Tribhuvan University Kathmandu Nepal; ^6^ Molecular Virology Laboratory School of Medicine University of Washington Seattle WA USA; ^7^ Harborview Medical Center University of Washington Seattle WA USA; ^8^ Department of Global Health Milken Institute School of Public Health George Washington University Washington DC USA

**Keywords:** Influenza, Low birth weight, Nepal, Pregnancy, Preterm, Small for gestational age, Vaccination

## Abstract

**Objective:**

To describe the effect of maternal vaccination on birth outcomes in rural Nepal, modified by timing of vaccination in pregnancy and influenza virus activity.

**Methods:**

A secondary analysis was conducted using data from two annual cohorts of a randomized controlled trial. A total of 3693 pregnant women from Sarlahi District were enrolled between April 25, 2011, and September 9, 2013. All participants were aged 15–40 years and received a trivalent inactivated influenza vaccine or placebo. The outcome measures included birth weight, pregnancy length, low birth weight (<2500 g), preterm birth, and small‐for‐gestational‐age birth.

**Results:**

Data were available on birth weight for 2741 births and on pregnancy length for 3623 births. Maternal vaccination increased mean birthweight by 42 g (95% confidence interval [CI] 8–76). The magnitude of this increase varied by season but was greatest among pregnancies with high influenza virus circulation during the third trimester. Birth weight increased by 111 g (95% CI −51 to 273) when 75%–100% of a pregnancy's third trimester had high influenza virus circulation versus 38 g (95% CI −6 to 81) when 0%–25% of a pregnancy's third trimester had high influenza virus circulation. However, these results were nonsignificant.

**Conclusion:**

Seasonal maternal influenza vaccination in rural Nepal increased birth weight; the magnitude appeared larger during periods of high influenza virus circulation.

**ClinicalTrials.gov:**

NCT01034254.

## Abstract 2

Maternal influenza vaccination in rural Nepal increased birth weight, particularly among pregnancies with high levels of influenza virus circulation during the third trimester.

## 1 INTRODUCTION

In 2010, influenza infection accounted for 19 200 000 disability‐adjusted life years worldwide.^1^ Pregnant women are at particularly high risk of complications from influenza infection.^2^ WHO recommends vaccination for pregnant women.^3^

One study showed markedly increased innate and adaptive immune responses to the influenza vaccine when comparing pregnant with non‐pregnant women,^4^ suggesting that these responses might underpin increased rates of morbidity and mortality among pregnant women. Furthermore, worse health outcomes resulting from influenza infection has been reported during late stages of pregnancy than during early stages.^5^ Maternal influenza infection has also been linked with fetal growth restriction, preterm delivery, and fetal loss.^6^

Neonates born growth restricted and/or preterm are likely to experience both immediate and long‐term health consequences. Those born small‐for‐gestational‐age (SGA)—a proxy for fetal growth restriction—experience increased risk of neonatal and infant mortality, stunted growth, and chronic disease in adulthood.^7^, ^8^ Likewise, those born preterm have increased risk of neonatal and infant mortality, as well as cognitive and motor impairments.^8^, ^9^

Observational studies and randomized controlled trials (RCTs) exploring the effect of maternal influenza vaccination on rates of SGA and preterm delivery have reported mixed findings.^10^, ^11^, ^12^, ^13^, ^14^, ^15^ The Nepal Mothers’ Gift Trial was an RCT of maternal influenza vaccination with two annual cohorts of pregnant women residing in a rural area of this country.^15^, ^16^ The primary outcomes were maternal influenza‐like illness, infant influenza infection, and low birth weight (LBW; defined as <2500 g). Inconsistent results were found between the two annual cohorts; however, protective effects were reported in at least one of these cohorts for all outcomes assessed.^15^

The aim of the present analysis was to present further details of the effect of maternal influenza vaccination on birth weight and pregnancy length among participants enrolled in the Nepal Mothers’ Gift Trial. The effect of maternal vaccination on birth outcomes and its modification by pregnancy length at which vaccination was given and by influenza virus activity were assessed. These issues are particularly important to address in locations with year‐round influenza circulation to determine the ideal timing of maternal vaccination.

## 2 MATERIALS AND METHODS

A secondary analysis was performed using the data from an RCT that examined the efficacy of influenza immunization on the incidence of respiratory illness and laboratory‐confirmed influenza infection among pregnant women and their neonates, with 6 months of postpartum follow‐up.^15^, ^16^ Ethical approval was obtained for the original study and subsequent analyses from the Institutional Review Boards of the Cincinnati Children's Medical Center (Cincinnati, OH, USA), Johns Hopkins Bloomberg School of Public Health (Baltimore, MD, USA), the Institute of Medicine at Tribhuvan University (Kathmandu, Nepal), and the Nepal Health Research Council (Kathmandu, Nepal). All participants provided informed consent to the original study and use of data for analysis.

Full details of the original methods and the results for the primary outcomes have been described elsewhere.^15^, ^16^ Briefly, married, pregnant women aged 15–40 years living in Sarlahi District, Nepal, were invited to participate. Women who had already received influenza vaccination that season, planned to deliver outside the study area, or had allergies to vaccine components (e.g. egg) were excluded. Eligible women were enrolled in two separate but sequential annual cohorts. For the first cohort, women at 17–34 weeks of pregnancy were enrolled between April 25, 2011, and April 24, 2012, and vaccinated on enrollment. For the second cohort, women were enrolled between April 25, 2012, and April 24, 2013; they were then randomly assigned a week between 17 and 34 weeks of pregnancy at which they would undergo vaccination. Pregnancy length was calculated from the date of the last menstrual period; recall of the date was aided by 5‐weekly population pregnancy surveillance by the study staff. Women in both cohorts were randomly allocated to receive a trivalent inactivated influenza vaccine or saline placebo in blocks of eight, stratified by pregnancy length at vaccination (17–25 or 26–34 weeks). The participants were masked to their allocation. Two different formulations of the vaccine were used (second formulation used starting from October 15, 2012). Details on the vaccines are available in Appendix S1.

All enrolled women were visited weekly by study staff, both during and after pregnancy (follow‐up continued to 180 days after delivery), to collect data on maternal and infant morbidities for each day in the previous week. These illness signs were all self‐reported by the women or other household members. Influenza‐like illness was defined as a fever in the presence of either a cough or sore throat, based on daily self‐reported data that was collected on a weekly basis. Nasal swabs were taken for influenza testing by polymerase chain reaction^17^ among all women presenting with fever plus one additional morbidity sign (persistent cough, sore throat, nasal congestion, or myalgia) and all infants with fever, cough, wheezing, difficulty in breathing, or otorrhea on any of the previous 7 days. Details of the laboratory tests are available elsewhere.^15^

The participants were instructed to notify study staff immediately after delivery, at which time a staff member visited the household to collect data on maternal, fetal, and neonatal status, as well as morbidities and conditions during labor and delivery.

The main outcome measures were birth weight and pregnancy length at delivery, and dichotomous variables LBW, preterm delivery (<37 weeks), and SGA. Only neonatal weights taken within 72 hours of delivery were included; when weight was taken beyond 72 hours, data on pregnancy length could still be contributed. A birth weight lower than the 10th percentile of the sex‐specific and gestational‐age‐specific INTERGROWTH‐21st birth weight standard^18^ was used to define SGA. Neonates at or above the 10th percentile were categorized as appropriate‐for‐gestational‐age (AGA).

The data were analyzed using Stata version 13 (StataCorp, College Station, TX, USA). The distribution of birth weight and pregnancy length at delivery was summarized by vaccination group. The data were also stratified by the formulation of the vaccine the mother received. The infants were categorized by creating mutually exclusive categories of delivery weight and pregnancy length as follows: term‐AGA‐not LBW, term‐AGA‐LBW, term‐SGA‐not‐LBW, term‐SGA‐LBW, preterm‐AGA‐not‐LBW, preterm‐AGA‐LBW, and preterm‐SGA (Fig. S1).^8^ Infants in the preterm‐SGA group were all LBW. The regression analyses conducted are summarized in Table 1.

**Table 1:** Description of analyses

Objective	Regression model	Independent variable(s) of interest	Dependent variable of interest	Stratified analyses
Determine association between maternal influenza or influenza‐like illness and birth outcome^a^	Adjusted linear regression^b^	1) Laboratory‐confirmed influenza at any point during pregnancy; 2) Influenza‐like illness at any point during pregnancy; 3) Total days of illness for laboratory‐confirmed influenza; 4) Total days of illness for influenza‐like illness	Birth weight	—
Association between influenza vaccination and birth outcome	1) Linear regression; 2) Log‐binomial regression; 3) Multinomial regression	Vaccination status (influenza vaccination or placebo)	1) Birth weight, pregnancy length as continuous outcomes 2) LBW, preterm, and SGA as binary outcomes 3) LBW‐preterm‐SGA categories as outcomes, using term‐AGA‐not LBW as the reference	• Stratified by 4‐mo groupings of calendar time the vaccination was provided, as there is seasonal variation in the birth outcomes • Stratified by maternal exposure to high, low, or no influenza circulation^c^ during the 3rd trimester (≥28 wk), given that the speed of fetal weight gain is greatest in the 3rd trimester • Stratified by whether the mother had 0 to <25%, 25 to <50%, 50 to <75%, or 75 to ≤100% of their 3rd trimester exposed to high circulation • Examined the interaction between exposure to high influenza circulation (percentage of 3rd trimester exposed to high influenza circulation) and vaccination and subsequently stratified the circulation analyses by when women were vaccinated in pregnancy (<26, 26 to <30, or ≥30 wk)

Abbreviations: LBW, low birth weight; SGA, small for gestational age; AGA, appropriate for gestational age.

a Analysis treated as observational study. Remaining analyses treated as randomized controlled trial.

b Adjusted for parity, maternal height, infant sex, seasonality of time of birth, and mother's age at first marriage.

c Each calendar week of the study period was categorized as high, low, or no influenza circulation. High circulation was defined as ≥0.25% of all enrolled mothers or infants contributing morbidity data that week testing positive for influenza, low circulation as >0% to <0.25%, and no circulation as 0%.

## 3 RESULTS

The flow diagrams for the two annual cohorts are shown in Figures S2 and S3. A total of 3693 pregnant women were enrolled; 1846 received placebo and 1847 received the influenza vaccine. The 17 deliveries that occurred within 2 weeks of the mothers receiving the vaccine were excluded from the present analysis on the assumption that immunologic protection after vaccination requires 2 weeks for full effect. The number of eligible live births was 1826 in the placebo group and 1820 in the vaccinated group. Birth weight was measured within 72 hours of delivery among 1361 (74.5%) women in the placebo group and 1380 (75.8%) in the vaccination group. Furthermore, 1813 (99.3%) women in the placebo group and 1810 (99.5%) in the vaccination group had a pregnancy length within the predefined feasibility range (24 to <50 weeks).

Among the 2741 women whose newborns were weighed within 72 hours of delivery, 31 (1.1%) had at least one laboratory‐confirmed influenza episode during pregnancy whereas 2710 (98.9%) did not. Additionally, 175 (6.4%) women experienced at least one episode of influenza‐like illness during pregnancy, whereas 2566 (93.6%) did not.

Table S1 presents birthweight stratified by whether the mother experienced influenza or influenza‐like illness. The adjusted regression results showed that the neonates of mothers with laboratory‐confirmed influenza at any point in pregnancy were on average 55 g (95% confidence interval [CI] −213 to 103) lighter at delivery than neonates whose mothers did not have influenza, but the difference was nonsignificant. Likewise, the neonates of mothers with influenza‐like illness at any point in pregnancy were significantly lighter at delivery than were those of mothers who did not experience influenza‐like illness (70 g, 95% CI −139 to −2). The total duration of illness was 1–26 days (median 4 days) for those with influenza and 1–12 days (median 3 days) for those with influenza‐like illness. The duration of such illness was not associated with birth weight (data not shown).

The birth weight distribution in the placebo and vaccination groups is presented in Table 2 and Figure S4. Greater differences in birth weight between the placebo and vaccination groups were seen during periods of more intensive A/H1N1 or B/Yamagata circulation (data not shown).

**Table 2:** Birth weight distribution, by vaccine type^a^ and vaccination status

Birth weight	Vaccine 1		Vaccine 2		Combined	
	Placebo (n=993)	Vaccine (n=1030)	Placebo (n=368)	Vaccine (n=350)	Placebo (n=1361)	Vaccine (n=1380)
Mean, g	2749	2785	2796	2859	2762	2804
Median, g	2760	2800	2790	2860	2770	2810
Interquartile range, g	2460–3040	2520–3080	2520–3075	2560–3150	2470–3050	2520–3100
Range, g	820–4420	1260–4110	1360–4140	1590–4800	820–4420	1260–4800
Low birth weight^b^	278 (28.0)	239 (23.2)	87 (23.6)	76 (21.7)	365 (26.8)	315 (22.8)

a Vaccine types are described in Appendix S1.

b Values given as number (percentage).

As previously reported,^15^ influenza vaccination increased mean birth weight by 42 g (95% CI 8–76) but had no effect on pregnancy length (difference 0.07 weeks, 95% CI −0.09 to 0.24). A statistically significant reduction was found for LBW (adjusted risk ratio 0.85, 95% CI 0.75–0.97) but not for SGA or preterm birth.^15^ The breakdown of neonates by SGA, preterm, and LBW is shown in Table 3. Compared with the placebo group, the vaccinated group had a greater proportion of infants in the term‐AGA category (the lowest mortality risk) and lower proportions in the high‐risk categories (term‐SGA‐LBW, preterm‐AGA‐LBW, and preterm‐SGA). The results of the regression analyses reflected these reductions, but were not statistically significant (Table 3).

**Table 3:** RRR of vaccination and categorical birth outcomes

Vaccination	Term‐AGA		Term‐SGA‐not LBW		Term‐SGA‐LBW		Preterm‐AGA‐not LBW		Preterm‐AGA‐LBW		Preterm‐SGA^a^	
	No. (%)	RRR	No. (%)	RRR	No. (%)	RRR	No. (%)	RRR	No. (%)	RRR	No. (%)	RRR
Vaccine 1												
Placebo (n=944)	461 (48.8)	Ref.	168 (17.8)	Ref.	180 (19.1)	Ref.	58 (6.1)	Ref.	59 (6.3)	Ref.	18 (1.9)	Ref.
Vaccinated (n=976)	512 (52.5)	Ref.	171 (17.5)	0.92 (0.72–1.17)	166 (17.0)	0.83 (0.65–1.06)	70 (7.2)	1.09 (0.75–1.57)	48 (4.9)	0.73 (0.49–1.09)	9 (0.9)	0.45 (0.20–1.01)
Vaccine 2												
Placebo (n=356)	193 (54.2)	Ref.	59 (16.6)	Ref.	61 (17.1)	Ref.	21 (5.9)	Ref.	17 (4.8)	Ref.	5 (1.4)	Ref.
Vaccinated (n=331)	187 (56.5)	Ref.	53 (16.0)	0.93 (0.61–1.41)	56 (16.9)	0.95 (0.63–1.43)	20 (6.0)	0.98 (0.52–1.87)	11 (3.3)	0.67 (0.30–1.46)	4 (1.2)	0.83 (0.22–3.12)
Combined												
Placebo (n=1300)	654 (50.3)	Ref.	227 (17.5)	Ref.	241 (18.5)	Ref.	79 (6.1)	Ref.	76 (5.9)	Ref.	23 (1.8)	Ref.
Vaccinated (n=1307)	699 (53.5)	Ref.	224 (17.1)	0.92 (0.75–1.14)	222 (17.0)	0.86 (0.70–1.06)	90 (6.9)	1.07 (0.77–1.47)	59 (4.5)	0.73 (0.51–1.04)	13 (1.0)	0.53 (0.27–1.05)

Abbreviations: RRR, relative risk ratio; AGA, appropriate for gestational age; LBW, low birth weight; SGA, small for gestational age.

a Preterm‐SGA infants were all LBW.

**Figure 1:**
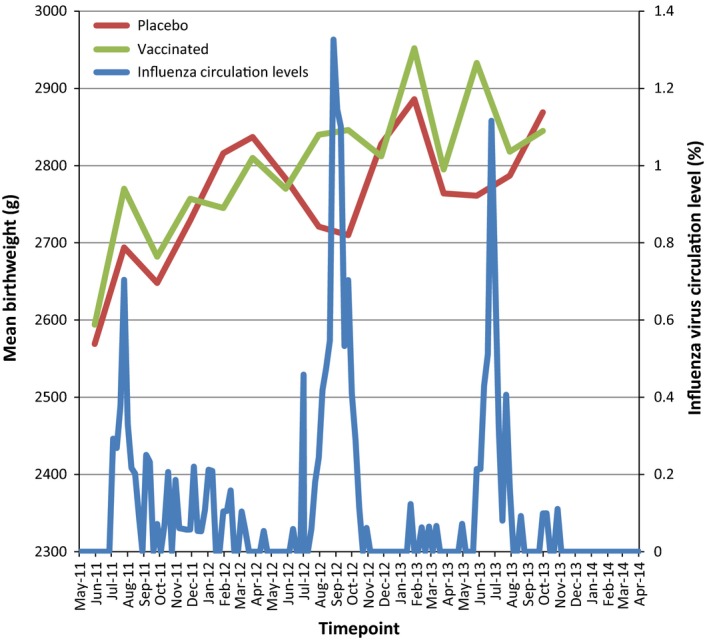
Mean birth weight in relation to influenza virus circulation.

Figure 1 displays the bimonthly mean birth weight in the placebo and vaccinated groups versus weekly influenza virus circulation. Figure S5 shows the same graph but with circulation levels stratified by the type of influenza virus. Generally, there were patterns of increased differences in birth weight, comparing vaccination with placebo, at times of high circulation for each viral strain.

With regard to secular trends, the mean birth weight increased by 5 g (95% CI 2–8) per month for the placebo group and 7 g (95% CI 4–10) per month for the vaccinated group through the course of the study.

Table S2 presents the effect of maternal vaccination on birth weight and pregnancy length, stratified by calendar time of vaccination (divided into 4‐month blocks owing to the sample size). Compared with women in the placebo group, birth weight was significantly higher among mothers vaccinated during January–April 2012 (mean difference 109 g, 95% CI 27–191), and during January–April 2013 (mean difference 112 g, 95% CI 12–212). A statistically significant reduction was seen for those vaccinated during September–December 2011 (mean difference −101 g, 95% CI −185 to −17). Among women vaccinated during May–August 2011, statistically significant reductions in LBW (RR 0.70, 95% CI 0.55–0.89) and SGA (RR 0.81, 95% CI 0.67–0.99) were seen. Statistically significant reductions in preterm births were found among those vaccinated during May–August 2012 (RR 0.55, 95% CI 0.33–0.92).

The vaccination effect on birth weight was stratified by exposure to high levels of influenza virus circulation in the third trimester (Table 4). Although none of the birth weight effects remained statistically significant, the data were indicative of a dose‐response pattern. Namely, the greater the proportion of the third trimester exposed to high influenza circulation, the greater the magnitude of birth weight gain associated with vaccination (Table 4). This dose‐response pattern could be observed when the analysis was limited to women who were vaccinated before 26 weeks, but the effects were not statistically significant (Table 4).

**Table 4:** The effect of vaccination on birthweight (in g), stratified by extent of third‐trimester exposure to high influenza circulation and timing of vaccination in pregnancy

Vaccination	Combined		Vaccinated at <26 wk		Vaccinated at 26–<30 wk		Vaccinated at ≥30 wk	
	n	β (95% CI)	n	β (95% CI)	n	β (95% CI)	n	β (95% CI)
0% to <25% of third trimester in high circulation period								
Placebo	1142	Ref.	779	Ref.	225	Ref.	138	Ref.
Vaccinated	1130	38 (−6 to 81)	766	42 (−11 to 95)	203	30 (−68 to 128)	161	39 (−85 to 163)
25% to <50% of third trimester in high circulation period								
Placebo	367	Ref.	244	Ref.	94	Ref.	29	Ref.
Vaccinated	384	21 (−49 to 90)	245	51 (−37 to 138)	114	−80 (−205 to 44)	25	109 (−136 to 353)
50% to <75% of third trimester in high circulation period								
Placebo	240	Ref.	136	Ref.	67	Ref.	37	Ref.
Vaccinated	236	66 (−32 to 165)	136	64 (−59 to 186)	64	32 (−157 to 222)	36	124 (−175 to 423)
75%–100% of third trimester in high circulation period								
Placebo	90	Ref.	43	Ref.	29	Ref.	18	Ref.
Vaccinated	86	111 (−51 to 273)	47	118 (−119 to 354)	24	304 (−14 to 622)	25	−86 (−397 to 225)
Combined								
Placebo	1845	Ref.	1206	Ref.	416	Ref.	223	Ref.
Vaccinated	1843	42 (8 to 76)	1191	48 (6 to 90)	405	23 (−47 to 93)	247	48 (−52 to 148)

Abbreviation: CI, confidence interval.

When stratifying by timing of vaccination in pregnancy, an increase in birth weight was seen in each category, but was only statistically significant among women vaccinated before 26 weeks (48 g, 95% CI 6–90) (Table 4).

A dose‐response relationship between exposure to high influenza circulation and pregnancy length was observed but was not statistically significant (Table S3). However, a statistically significant increase in pregnancy length was found among mothers who were vaccinated between 26 weeks and less than 30 weeks, when at least half of their third trimester had been exposed to high influenza virus circulation. When removing the circulation stratification, there was a statistically significant positive effect on pregnancy length among those vaccinated between 26 weeks and less than 30 weeks, but not for those vaccinated before or after that time (Table S3).

When the proportion of a pregnancy's third trimester exposed to high influenza virus circulation was examined as a continuous variable, vaccination during periods of greater influenza virus circulation had a greater positive effect on birth weight than vaccination during periods of lower influenza virus circulation, but the effect modification was not statistically significant. A positive trend of increased pregnancy length was observed among vaccinated individuals with greater third trimester exposure to influenza circulation than with lower third trimester exposure; however, the effect modification was not statistically significant (data not shown).

## 4 DISCUSSION

Maternal influenza vaccination increased birth weight among live births in the present study. The magnitude differed by season, appearing greatest among pregnancies for which the third trimester occurred at a time of high influenza virus circulation; however, the results were inconclusive. Sarlahi District in southern Nepal is a subtropical region that experiences influenza virus circulation for two‐thirds of the year and is epidemiologically similar to other parts of South Asia. Seasonal peaks of circulation appear to overlap temporally with an increased effect of vaccination on birth weight. In a low‐resource setting such as rural Nepal, vaccination delivery can be logistically difficult; therefore, alternatives to year‐round vaccination should be considered. The present study also indicated a marginal reduction in the prevalence of infants born both preterm and SGA. No clear trends were observed to link timing of vaccination during pregnancy with the effects on birth weight.

Previous observational studies reported mixed results regarding the effect of maternal influenza vaccination on birth outcomes. Data collected in the USA between 2004 and 2009 showed no effect on preterm birth or SGA,^19^ although rates of these outcomes are much lower in the USA than in low‐income countries. Data from the 2009 US H1N1 influenza pandemic showed that preterm rates were 37% lower (adjusted odds ratio 0.63, 95% CI 0.47–0.84) for the vaccinated versus the non‐vaccinated, but there was a minimal birth weight effect and no change in SGA or LBW.^20^ Although these observational studies adjusted for confounders, residual confounding is likely because vaccinated women likely differ in characteristics from unvaccinated women.

Four maternal influenza vaccination trials have been conducted to date, including the Nepal Mothers’ Gift Trial.^15^, ^16^ A Bangladeshi study found only a statistically significant increase in birth weight and a decrease in SGA rate during the high influenza virus circulation period.^14^ Studies conducted in South Africa^13^ and Mali^12^ found no effect on birth weight or LBW, irrespective of influenza virus circulation. The consistency of the present findings with the Bangladeshi study^14^ could imply a regional difference. The LBW and SGA rates are substantially higher in Asia than in Sub‐Saharan Africa,^21^ which might indicate varying etiological factors that affect birth weight. An outcome of LBW, which is comprised of preterm delivery and/or fetal growth restriction, can result from various maternal exposures, including nutrition, substance misuse, environment, and infection.^21^ Rates of malnutrition are high in Asia; therefore, it is possible that poor nutrition aggravates the adverse effect of infectious disease. There is extensive evidence linking malnutrition with increased susceptibility to, and/or severity of, infection.^22^ A study conducted in the Democratic Republic of Congo^23^ showed that the association between malaria and fetal growth restriction was only present if women presented with poor nutritional indicators, such as low body mass index and low mid‐upper arm circumference. The present study did not capture sufficient cases of influenza or influenza‐like illness to stratify the analyses by maternal nutrition status.

The present study examined only the effect of vaccination on birth weight and pregnancy length; however, there is evidence to suggest that influenza vaccination might positively impact fetal and neonatal health outcomes through other mechanisms. Animal studies have indicated effects of maternal influenza infection on fetal brain development,^24^ and an analysis of the 1918 influenza pandemic found excess risk of cardiovascular disease among individuals born during this pandemic.^25^ The present study found a substantial reduction in preterm‐SGA incidence among vaccinated mothers. Preterm‐SGA infants experience the highest risk of neonatal and postneonatal mortality. Asian data that were comprised predominantly of South Asian studies showed a 17‐fold increase in neonatal mortality risk when comparing preterm‐SGA with term‐AGA infants.^8^ Rates of stillbirth have also been reported to be higher among growth‐restricted and/or preterm fetuses.^26^ Although the present sample size was not large enough to detect differences in neonatal mortality, a protective effect against mortality might be expected with the reduction in the proportion of preterm‐SGA infants.

The present study could not determine what period in pregnancy would be associated with the largest effect from exposure to influenza virus. Given that the greatest fetal weight gain occurs late in pregnancy,^27^ the present study focused on such exposure during the third trimester; however, exposure at an earlier stage in pregnancy could trigger inflammatory responses that affect other health outcomes. In attempting to understand the interaction between seasonality of influenza virus circulation and the stages of pregnancy, the present study could not definitively identify an ideal time for maternal influenza vaccination. Additionally, the viral surveillance performed might have been limited because nasal swabs for influenza testing were taken only among women who reported a febrile respiratory illness.

In conclusion, maternal influenza vaccination exerted the greatest effect on birth weight among pregnant women who were exposed to high influenza virus circulation during the third trimester. A strategy of seasonal maternal influenza vaccination in resource‐limited settings could potentially increase birth weight during periods of high influenza circulation. Taking into consideration the barriers to providing year‐round vaccination, it may be possible to target key times of the year to maximize the impact on birth outcomes.

## AUTHOR CONTRIBUTIONS

NK conducted the data analysis and wrote the first draft of the manuscript. JKa, JAE, MCS, SKK, LS, JKu, LCM, and JMT contributed to the study design. JKa, SKK, LS, LCM, SCL, and JMT supervised the conduct of the study in the field. JAE and JKu determined the virology assay collection protocols. HYC assisted in study implementation and cord‐blood collection. JAE and MCS determined the antibody assay protocols. All authors contributed to the writing or revisions to the manuscript and approved the final version.

## CONFLICTS OF INTEREST

JKa, MCS, LCM, HYC, and JMT have received grants from the Bill & Melinda Gates Foundation. JAE has received grants and personal fees from GlaxoSmith Kline, Pfizer, and Gilead; research support from Roche, Novavax, Chimerix, MedImmune, and Alio; and personal fees from Abbvie. MCS has received funding from Pfizer. HYC has received grants from PATH and the Thrasher Foundation, and research funding from Sanofi, GlaxoSmith Kline, and Novavax. The other authors have no conflicts of interests.

## Supporting information


**Figure S1.** Categorical breakdown of birth weight and pregnancy length.

**Figure S2.** Flow of patients through the first cohort.

**Figure S3.** Flow of patients through the second cohort.

**Figure S4.** Birth weight distribution by vaccine status and vaccine type.

**Figure S5.** Bimonthly mean birth weight against weekly influenza circulation, by influenza type.

**Table S1.** Birth weight distribution (restricted to those measured within 72 hours of birth), comparing women who had influenza or influenza‐like illness during pregnancy with those who did not.

**Table S2.** Effect of maternal vaccination on birth outcomes, stratified by calendar time of vaccination.

**Table S3.** Effect of vaccination on gestational age, stratified by third trimester exposure to high influenza circulation and timing of vaccination in pregnancy.

**Appendix S1.** Influenza vaccine type.
