# Supplementary figures and images for: Impact of maternal vaccination timing and influenza virus circulation on birth outcomes in rural Nepal
Source: Int J Gynaecol Obstet. 2017 Nov 9;140(1):65–72. doi: 10.1002/ijgo.12341 (PMC5765513; doi:10.1002/ijgo.12341)

**
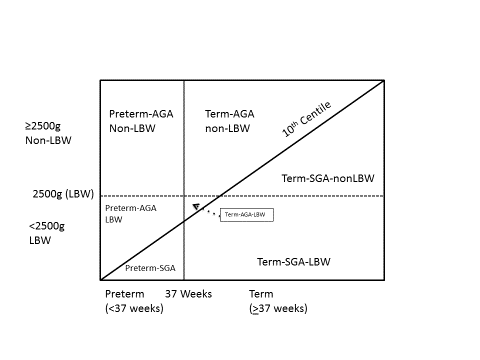
**

Supplement: Supplementary file 1 — Figure S1. Categorical breakdown of birth weight and pregnancy length. [file IJGO-140-65-s001.docx]

**
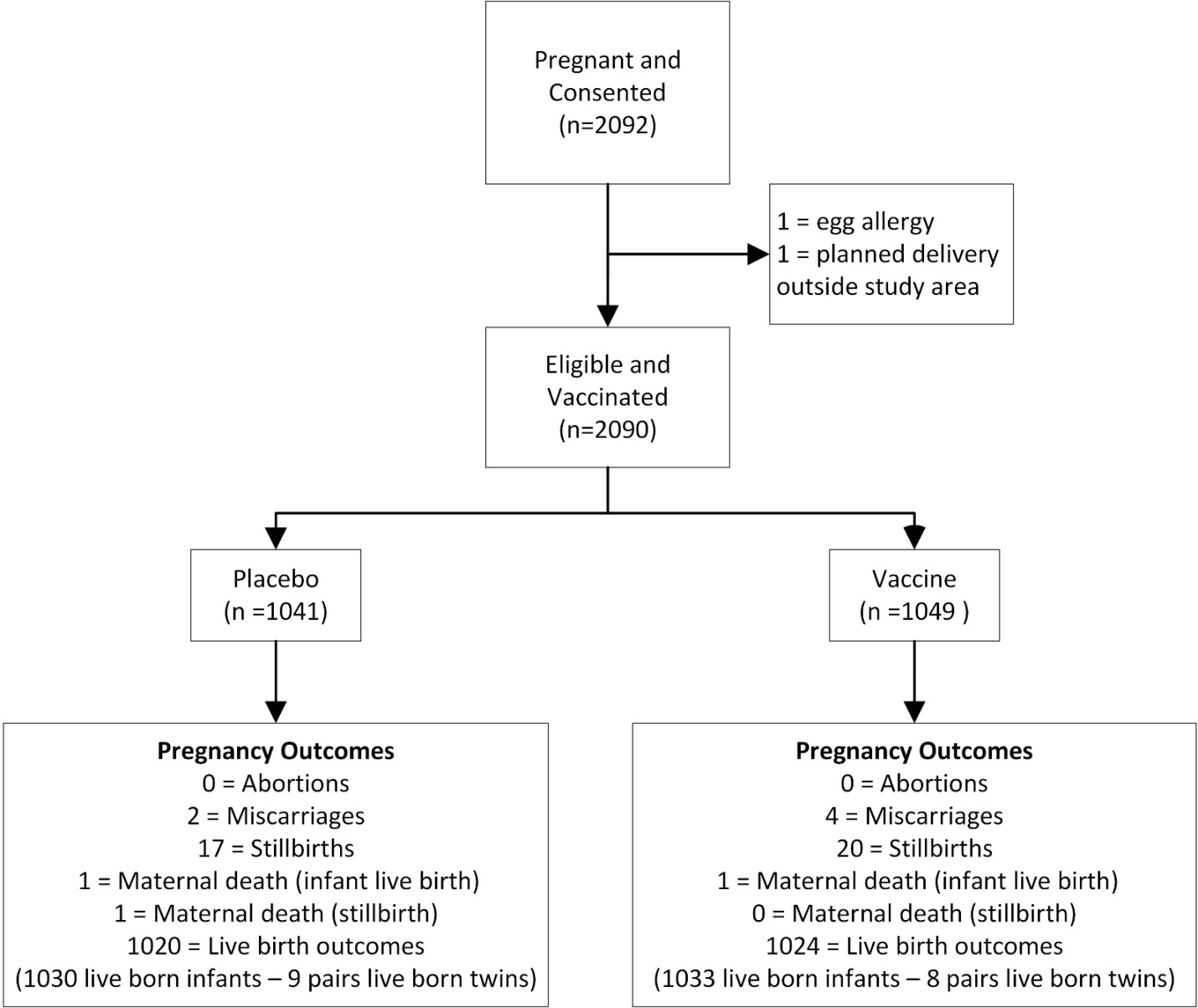
**

Supplement: Supplementary file 2 — Figure S2. Flow of patients through the first cohort. [file IJGO-140-65-s002.docx]

**
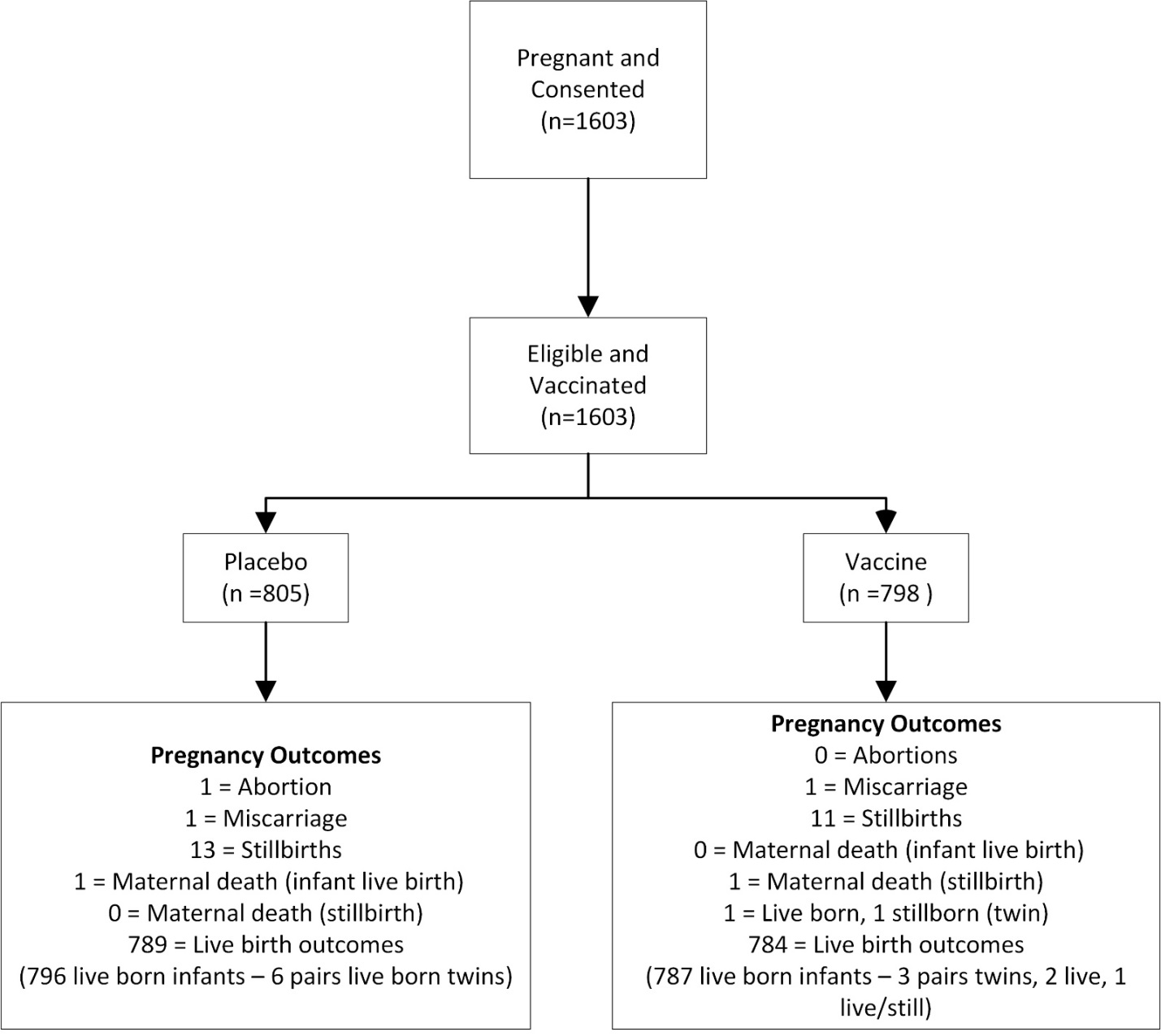
**

Supplement: Supplementary file 3 — Figure S3. Flow of patients through the second cohort. [file IJGO-140-65-s003.docx]
