# Supplementary material for: Impact of maternal vaccination timing and influenza virus circulation on birth outcomes in rural Nepal
Source: Int J Gynaecol Obstet. 2017 Nov 9;140(1):65–72. doi: 10.1002/ijgo.12341 (PMC5765513; doi:10.1002/ijgo.12341)
Supplement: Supplementary file 4 — Figure S4. Birth weight distribution by vaccine status and vaccine type. [file IJGO-140-65-s004.docx]

*Vaccine 1 was administered between April 25, 2011 and October 14, 2012, and contained the Perth A/H3N2, California A/H1N1, and Brisbane (Victoria) B strains. Vaccine 2 was administered between October 15, 2012 and September 9, 2013, and contained the Victoria A/H3N2, California A/H1N1 and Wisconsin (Yamagata) B strains.
