# Supplementary material for: Impact of maternal vaccination timing and influenza virus circulation on birth outcomes in rural Nepal
Source: Int J Gynaecol Obstet. 2017 Nov 9;140(1):65–72. doi: 10.1002/ijgo.12341 (PMC5765513; doi:10.1002/ijgo.12341)
Supplement: Supplementary file 5 — Figure S5. Bimonthly mean birth weight against weekly influenza circulation, by influenza type. [file IJGO-140-65-s005.docx]

**Figure S5A** Bimonthly mean birthweight against weekly influenza circulation, by influenza type

**Figure S5B** Bimonthly mean birthweight against weekly A (H3N2) circulation

**Figure S5C** Bimonthly mean birthweight against weekly A (H1N1) circulation

**Figure S5D** Bimonthly mean birthweight against weekly A (not typed) circulation

**Figure S5E** Bimonthly mean birthweight against weekly B (Victoria) circulation

**Figure S5F** Bimonthly mean birthweight against weekly B (Yamagata) circulation

**Figure S5G** Bimonthly mean birthweight against weekly B (not typed) circulation
