# Supplementary material for: Impact of maternal vaccination timing and influenza virus circulation on birth outcomes in rural Nepal
Source: Int J Gynaecol Obstet. 2017 Nov 9;140(1):65–72. doi: 10.1002/ijgo.12341 (PMC5765513; doi:10.1002/ijgo.12341)
Supplement: Supplementary file 6 — Table S1. Birth weight distribution (restricted to those measured within 72 hours of birth), comparing women who had influenza or influenza‐like illness during pregnancy with those who did not. [file IJGO-140-65-s006.docx]

**Table S1** Birth weight distribution (restricted to those taken within 72 hours of birth), comparing women who had influenza or influenza-like illness during pregnancy with those who did not.

|  | **Lab-confirmed Influenza** | | **Influenza-like illness (ILI)** | |
| --- | --- | --- | --- | --- |
|  | **Had flu (n=31)** | **No flu (n=2710)** | **Had ILI (n=175)** | **No ILI (n=2566)** |
| mean | 2697 | 2784 | 2708 | 2788 |
| median | 2720 | 2800 | 2700 | 2800 |
| IQR | 2350-2860 | 2500-3080 | 2380-2990 | 2500-3080 |
| range | 1560-4005 | 820-4800 | 1530-4080 | 820-4800 |
