# Supplementary material for: Impact of maternal vaccination timing and influenza virus circulation on birth outcomes in rural Nepal
Source: Int J Gynaecol Obstet. 2017 Nov 9;140(1):65–72. doi: 10.1002/ijgo.12341 (PMC5765513; doi:10.1002/ijgo.12341)
Supplement: Supplementary file 7 — Table S2. Effect of maternal vaccination on birth outcomes, stratified by calendar time of vaccination. [file IJGO-140-65-s007.docx]

**Table S2** Effect of maternal vaccination on birth outcomes, stratified by calendar time of vaccination

|  | **Birth weight** | | **Gestational age** | | **LBW** | | **Preterm** | | **SGA** | |
| --- | --- | --- | --- | --- | --- | --- | --- | --- | --- | --- |
| **Timing of vaccination** | **n** | **β (95% CI)** | **n** | **β (95% CI)** | **n** | **RR (95% CI)** | **n** | **RR (95% CI)** | **n** | **RR (95% CI)** |
| April 2011 | 73 | -35.4 (-221.9, 151.1) | 87 | 0.01 (-1.00, 1.02) | 73 | 1.17 (0.48, 2.90) | 87 | 1.95 (0.38, 10.12) | 71 | 1.03 (0.58, 1.83) |
| May-Aug | 695 | 64.5 (-6.2, 135.3) | 906 | 0.27 (-0.06, 0.61) | 695 | 0.70 (0.55, 0.89) | 906 | 0.79 (0.57, 1.10) | 655 | 0.81 (0.67, 0.99) |
| Sep-Dec | 419 | -100.6 (-184.6, -16.6) | 528 | -0.47 (-0.96, 0.01) | 419 | 1.33 (0.95, 1.86) | 528 | 1.26 (0.83, 1.89) | 402 | 1.07 (0.83, 1.38) |
| Jan-Apr 2012 | 412 | 109.1 (27.2, 190.9) | 548 | -0.06 (-0.51, 0.38) | 412 | 0.75 (0.53, 1.06) | 548 | 0.94 (0.61, 1.43) | 388 | 0.83 (0.64, 1.08) |
| May-Aug | 312 | 77.9 (-16.8, 172.7) | 450 | 0.40 (-0.04, 0.84) | 312 | 0.70 (0.47, 1.04) | 450 | 0.55 (0.33, 0.92) | 299 | 1.04 (0.75, 1.44) |
| Sep-Dec | 348 | 18.9 (-76.8, 114.6) | 464 | -0.15 (-0.60, 0.31) | 348 | 1.27 (0.83, 1.95) | 464 | 1.40 (0.88, 2.23) | 332 | 0.92 (0.67, 1.26) |
| Jan-Apr 2013 | 341 | 112.0 (12.0, 212.0) | 444 | 0.27 (-0.16, 0.70) | 341 | 0.69 (0.47, 1.03) | 444 | 0.91 (0.54, 1.53) | 325 | 1.08 (0.82, 1.43) |
| May-Aug | 141 | 15.1 (-130.9, 161.1) | 196 | 0.18 (-0.48, 0.83) | 141 | 0.89 (0.51, 1.55) | 196 | 0.48 (0.19, 1.21) | 135 | 0.90 (0.55, 1.46) |
