# Supplementary material for: Impact of maternal vaccination timing and influenza virus circulation on birth outcomes in rural Nepal
Source: Int J Gynaecol Obstet. 2017 Nov 9;140(1):65–72. doi: 10.1002/ijgo.12341 (PMC5765513; doi:10.1002/ijgo.12341)
Supplement: Supplementary file 8 — Table S3. Effect of vaccination on gestational age, stratified by third trimester exposure to high influenza circulation and timing of vaccination in pregnancy. [file IJGO-140-65-s008.docx]

**Table S3** The effect of vaccination on gestational age, stratified by third trimester exposure to high influenza circulation and timing of vaccination in pregnancy.

|  | **Combined** | | **Vaccinated <26 weeks gestation** | | **Vaccinated 26-<30 weeks gestation** | | **Vaccinated >=30 weeks gestation** | |
| --- | --- | --- | --- | --- | --- | --- | --- | --- |
|  | **n** | **β (95% CI)** | **n** | **β (95% CI)** | **n** | **β (95% CI)** | **n** | **β (95% CI)** |
| 0-<25% of third trimester in high circulation period | | | | | | | | |
| Placebo | 1142 | Ref | 779 | Ref | 225 | Ref | 138 | Ref |
| Vaccinated | 1130 | -0.01 (-0.22, 0.20) | 766 | -0.03 (-0.29, 0.23) | 203 | 0.38 (-0.09, 0.86) | 161 | -0.52 (-1.02, -0.03) |
| 25-<50% of third trimester in high circulation period | | | | | | | | |
| Placebo | 367 | Ref | 244 | Ref | 94 | Ref | 29 | Ref |
| Vaccinated | 384 | 0.03 (-0.28, 0.35) | 245 | -0.09 (-0.46, 0.29) | 114 | 0.01 (-0.60, 0.63) | 25 | 1.07 (-0.16, 2.29) |
| 50-<75% of third trimester in high circulation period | | | | | | | | |
| Placebo | 240 | Ref | 136 | Ref | 67 | Ref | 37 | Ref |
| Vaccinated | 236 | 0.17 (-0.23, 0.57) | 136 | -0.15 (-0.70, 0.40) | 64 | 0.88 (0.21, 1.56) | 36 | 0.13 (-0.81, 1.08) |
| 75-100% of third trimester in high circulation period | | | | | | | | |
| Placebo | 90 | Ref | 43 | Ref | 29 | Ref | 18 | Ref |
| Vaccinated | 86 | 0.74 (-0.02, 1.51) | 37 | 0.83 (-0.39, 2.05) | 24 | 0.80 (-0.60, 2.19) | 25 | 0.33 (-1.03, 1.69) |
| All mothers | | | | | | | | |
| Placebo | 1845 | Ref | 1206 | Ref | 416 | Ref | 223 | Ref |
| Vaccinated | 1843 | 0.07 (-0.09, 0.23) | 1191 | -0.02 (-0.22, 0.19) | 405 | 0.48 (0.15, 0.82) | 247 | -0.23 (-0.64, 0.18) |
